# How Much Participant Outcome Data Is Missing from Sight: Findings from a Cohort of Trials Submitted to a German Research Ethics Committee

**DOI:** 10.1371/journal.pone.0157883

**Published:** 2016-06-17

**Authors:** Jamie J. Kirkham, Kerry M. Dwan, Anette Blümle, Erik von Elm, Paula R. Williamson

**Affiliations:** 1 MRC North West Hub for Trials Methodology Research, Department of Biostatistics, University of Liverpool, Liverpool, United Kingdom; 2 Cochrane Editorial Unit, London, United Kingdom; 3 Cochrane Germany, Faculty of Medicine, University of Freiburg, Freiburg, Germany; 4 Cochrane Switzerland, Institut Universitaire de Médecine Sociale et Préventive, Lausanne, Switzerland; University of Vienna, AUSTRIA

## Abstract

**Background:**

Study publication bias and outcome reporting bias have been recognised as two threats to the validity of systematic reviews. The purpose of this research was to estimate the proportion of missing participant outcome data from randomised controlled trials (RCTs) due to lack of publication of whole studies and due to outcome data missing within study publications.

**Methods and Findings:**

Data were extracted from protocols of clinical research projects submitted to the research ethics committee of the University of Freiburg (Germany) between 2000 and 2002 and associated fully published articles. The total amount of published and unpublished outcome data from all trial participants was calculated for each trial and the overall proportion of missing data from both unpublished and published trials computed. Full and partially reported outcome data was also taken into consideration. The impact of funding source on missingness was also considered at the trial level. From 308 parallel group trials in the study cohort, 167 were published and 141 were unpublished. Overall, 260,563 participants contributed to a total of 2,618,116 participant outcome data across all trials. About half (47%) of the participant outcome data from the 308 trials was reported in full but at least 81% were partially reported. Of the 19% of participant data that were missing, 4% was attributable to missing data from published trials and 15% from unpublished trials. Commercially funded trials had a higher probability of publication (relative risk 1.20, 95% confidence interval 0.86, 1.67; p = 0.27) but were less likely to fully report all outcomes than non-commercially funded trials (relative risk 0.64, 95% confidence interval 0.30, 1.38; p = 0.26).

**Conclusions:**

Missing participant outcome data from both published and unpublished trials is frequent. Clinical trial registration including outcome information not only identifies that clinical trials exist but the systematic examination and monitoring of trial information within a registry can help detect selective reporting of entire studies and of outcome data within studies and possibly prevent it.

## Background

Study publication bias and within-study outcome reporting bias have been recognised as two forms of reporting bias that can affect evidence based practice. There is strong evidence of an association between direction of results and publication status; studies that report positive or significant results are more likely to be published and outcomes that are statistically significant have higher odds of being fully reported (range of odds ratios: 2.2 to 4.7) [1,2]. As a result of these two forms of bias, systematic reviews summarising the available evidence are likely to overestimate the effect of treatment [3,4]. Reporting bias may therefore result in inappropriate health care decisions by policy makers, clinicians and patients, which potentially harm patients, waste resources, and misguide future research. While there is growing empirical evidence for the existence of these biases, little is known about the relative size of these two related problems affecting the available research evidence. Using data taken from protocols of clinical research projects submitted to the research ethics committee of the University of Freiburg (Germany) and associated fully published articles, we estimate the proportion of missing participant outcome data due to lack of publication of the study and the proportion due to missing outcome data within a published study.

## Methods

### Data source

The data used in this study has previously been identified and evaluated in other publications [5–7]. In brief, the Research Ethics Committee (REC) of the Albert-Ludwigs-University, Freiburg, Germany granted access to the electronic files of all study protocols (including amendments) submitted between 2000 and 2002. Nearly half (408/917; 44%) of the studies approved by the REC Freiburg within this time period were randomised controlled trials (RCTs). Of the 355 RCTs that started, the majority were multicentre trials and in 12%, the primary centre was located in Freiburg. In this research article we consider only the 311 RCTs with parallel group design that started. Trials that never started were excluded since there was no expectation that the trial results would be published. Full study publications were identified through a systematic search of electronic literature databases and verification via contact with the trial authors. Unpublished trials were defined to be trials where it was confirmed by trial authors that the trial was completed or discontinued and there was no full study publication. To ensure that the current dataset was as up-to-date as possible, the publication status of ongoing trials (at the time of the original study [5]) was verified again in July 2015. This enabled us to classify all trials as either published or unpublished.

### Data extraction

The total number of trials in the study cohort was separated into those that were published and those that were unpublished. For published trials, the total number of participants was extracted from the trial publication which represented the actual sample size achieved (i.e. the total number randomised), the largest of which was taken if sample sizes differed across multiple publications of the same trial. For unpublished trials, as an approximation to what was actually collected (but not published), planned sample sizes were extracted from the trial protocol. The sample size was assumed to be the same for all trial outcomes. For each trial, published or unpublished, we also extracted the total number of pre-specified outcomes listed in the trial protocol. For published trials, we recorded the total number of published and unpublished outcomes taken from the trial publication.

Published outcomes were categorised into ‘full’ reporting and ‘partial’ reporting. An outcome was considered to be fully reported if sufficient information was provided such that the outcome could be included in a potential meta-analysis of a systematic review in accordance to the definitions provided in [8]. Partially reported outcomes were defined as those that were inadequately reported for inclusion into a review meta-analysis (for example, an effect size was presented with no measure of precision or exact p-value). An extended list of the different types of partial reporting can be found in the data supplement (Web table B) of the main ORBIT (Outcome Reporting Bias in Trials) manuscript [3]. The highest order of reporting was taken for each outcome if multiple time-points were considered, for example, if an outcome was partially reported at 3 and 6 months and fully reported at 12 months, then the outcomes was considered to be fully reported. The source of funding was also extracted for each trial. Funding source was categorised into commercially funded (typically funded by pharmaceutical industry), non-commercially funded (typically funded by research councils, research foundations, government ministries and charities) and trials where no source of funding was declared. Trials were classified as commercially funded if they were partially funded by industry but were categorised as non-commercially funded if a company provided the trial drugs (wholly or in part) but had no role in the study design, data collection and analysis.

Data extraction from the original protocols and publications was undertaken in Freiburg in which AB contributed. Using the database of extracted data, the required information for this trial was data extracted independently by two researchers (KD and JJK). Any discrepancies between the information extracted by the two reviewers were resolved through discussion and in consultation with AB. Sample sizes and a summary of the outcome status’ (fully reported, unreported and partially reported) for each individual trial can be found in the supporting information file (S1 File).

### Data analyses

#### Total amount of reported and missing participant outcome data (participant data level)

The total amount of participant outcome data from each trial was calculated to be the planned trial sample size multiplied by the total number of pre-specified outcomes. For the study cohort, we computed the total amount of participant data that was fully reported, partially reported and missing from the published trials and the total amount of data that was missing as a result of trials being unpublished. Using the computed metrics summed across all trials and taking the total amount of outcome data across all trials as the denominator, we report the following proportions:

- Proportion of fully published data
- Proportion of partially reported data
- Proportion of fully and partially reported data
- Proportion of missing data from published trials
- Proportion of missing data from unpublished trials
- Proportion of missing data from all trials (published and unpublished).

Missing data from published trials may be due to within study selective reporting. In our computations, we also considered partially reported outcome data as unpublished data. The motivation here is that, although partially reported outcome data are published, they cannot be included into a meta-analysis without obtaining further information (which is usually only available from the authors), and therefore effectively resembles missing data in the systematic review process.

#### Impact of funding source (trial level)

The relative risk of full publication was compared between commercially funded trials and non-commercially funded trials. For the published trials, we also computed the relative risk of fully reporting all outcomes in commercially funded trials compared to non-commercially funded trials.

## Results

The study cohort contained 311 RCTs of which three were excluded as they presented no results for the comparator group. This is a high risk of bias form of selective reporting which has previously been documented [3], and our decision to exclude here is that our missing data computations would mask this serious reporting problem by simply reducing the sample sizes of these trials to what was either reported in the treatment arm or not reported in the comparator arm. Of the remaining 308 trials, 167 were published (54%) and 141 (46%) were unpublished. There was a tendency for published trials to specify more outcomes in their protocols than unpublished trials (Table 1). Further, the median number of recruited participants was higher than the number of planned participants in unpublished trials, and a higher proportion of published trials were commercially funded (Table 1). The majority of trials (published and unpublished) were multi-centre trials and of these multi-centre trials, most were international trials (Table 1). The primary centre for the multi-centre trials was Freiberg for 12% of published trials and 14% for unpublished trials.

**Table 1:** Outcome, participant and funding characteristics of trials that were published and unpublished.

	All	Published	Unpublished
	n = 308 (%)^a^	n = 167 (%)^a^	n = 141(%)^a^
**Outcomes**			
Total number of outcomes:	3407	2253	1154
Median (per trial):	10	11	7
Interquartile range:	5 to 15	7 to 17	4 to 11
**Trial participants**			
Total number of participants:	260,563	210,191	50,372
Median (per trial):	200	210	180
Interquartile range:	80 to 463	87 to 62	78 to 320
**Funding**			
Commercial:	198 (64)	119 (71)	79 (56)
Non-commercial:	40 (13)	20 (12)	20 (14)
Not stated:	70 (23)	28 (17)	42 (30)
**Collaboration**			
Single-centre:	65 (21)	28 (17)	37 (26)
Multi-centre:	243 (79)	139 (83)	104 (74)
**Only multi-centre studies**^b^			
International:	162 (67)	107 (77)	55 (53)
National:	80 (33)	32 (23)	48 (46)
Unclear:	1 (<1)	0	1 (1)
**Primary centre**^b^			
Freiburg:	31 (13)	16 (12)	15 (14)
Other:	211 (87)	123 (88)	88 (85)
Unclear:	1 (<1)	0	1 (1)

^a^column percentages;

^b^denominator is number of multi-centre studies

The 308 published and unpublished trials contained data on 3407 distinct outcomes of 260,563 participants (Table 1). From the published trials 70% (1595/2253) of the outcomes were published in full, 7% (167/2253) of the outcomes went unreported while the remainder were partially reported. Clearly all 1154 outcomes from the unpublished studies went unreported.

At the individual participant level, the total amount of participant outcome data was 2,618,116 (Table 2). Under a half of this data (47%) was fully reported but at least 81% were partially reported. Four percent of the data was missing (within-study selective outcome reporting) from published trials and 15% was missing from entirely unpublished trials.

**Table 2:** Proportion of reported and missing participant outcome data.

**1) Type of data (source)**	**Total amount**	
Participant data (published and unpublished studies)	2,618,116	[A]
Fully reported data (published studies)	1,233,291	[B]
Partially reported data (published studies)	892,892	[C]
Missing data (published studies)	91,381	[D]
Unpublished data (unpublished studies)	400,552	[E]
**2) Proportions of total amount of outcome data**		
Fully published data	47%	[B]/[A]
Partially reported data	34%	[C]/[A]
Proportion of fully and partially reported data	81%	[B+C]/[A]
Missing data from published studies (*within-study selective outcome reporting*)	4%	[D]/[A]
Missing data from unpublished studies (*selective reporting of entire studies*)	15%	[E]/[A]
Missing data from all studies	19%	[D+E]/[A]
Missing data from all studies (when partially reported was considered as unpublished data)	53%	[C+D+E]/[A]

Sixty percent (119/198) of commercially funded trials were published while 50% (20/40) of non-commercially funded trials were published. A commercially funded trial had a 20% increased probability of being published compared to a non-commercially funded trial although this difference did not reach statistical significance (relative risk 1.20, 95% confidence interval (0.86, 1.67; p = 0.27)). This wide confidence interval suggested that there was a high level of uncertainty in this finding.

Of the published trials, nearly a fifth (23/119; 19%) of the commercially funded trials fully reported all pre-specified outcomes compared to 30% (6/20) of the non-commercially funded trials. Commercially funded trials were less likely to fully report all outcomes than non-commercially funded trials although this difference again did not reach statistical significance with a wide confidence interval denoting a high level of uncertainty in this finding (relative risk 0.64, 95% confidence interval (0.30, 1.38; p = 0.26)).

## Discussion

Our study has shown that (81%) of participant outcome data from trials is either reported in full or partially reported. Direct empirical evidence for the existence of study publication bias and outcome reporting bias has been summarized previously but [1], to our knowledge, this study is novel seeking to quantify how much outcome data is missing from trials and how much of this missingness is attributable to lack of publication of entire studies or of outcomes from studies for which other data has been published. Missing data from published studies which could be associated with within-study selective outcome reporting appeared to be less of a problem than missing data from entirely unpublished studies. Nevertheless we have shown that the amount of missing data from both published and unpublished trials is more than half (53%) if partially reported data is treated as unpublished. Considering partially reported data as unpublished reflects the fact that such data cannot be included in a meta-analysis right away. In a survey, most Cochrane review authors related that they requested additional unpublished data from trialists but only about half of them obtain them [9]. Furthermore, partial reporting has previously been associated with a high risk of outcome reporting bias [3,10]. Our study is also likely to underestimate the impact of partial reporting, since outcomes with multiple time-points were considered to be fully reported if just one time-point was reported in full.

### Strengths and limitations of this study

We evaluated a large, unselected cohort of trials, the investigators confirmed whether or not the trial was published if no trial publication was found by us, and two study authors independently extracted and verified all the trial data. A limitation of our study is that we assumed that the sample sizes from the published reports were the same for all trial outcomes. This assumption may not always be realistic and may conflate attrition with the failure to report. However, sample sizes for individual outcomes are not routinely reported for all trials thus our approach could be consistently applied across all trials. All the trials included in the study were identified from protocols submitted to a German REC and hence some of the final trial publications were subsequently published in German language journals. It is possible that the results may therefore not fully represent cohorts of trials from other RECs. Previously, we identified four additional studies that covered the entire process from the publication of the study protocol to the publication of the study outcomes that could have also been used as potential data sources for comparison [1]. However, at the time of this original review, these datasets were unavailable after several requests were made to the study authors and it remains unclear as to whether these studies extracted data on sample sizes needed for inclusion into this current research. These other studies were approved by the Canadian Institute of Health Research (Canada) [11], the Scientific-Ethical Committees for Copenhagen and Frederiksberge (Denmark) [8], Central Sydney Area Health Service Ethics Review Committee (Australia) [12] and Local Research Ethics Committee (UK) [13].

## Conclusions

The full publication of outcome data from clinical trials helps avoid unnecessary duplication of research while clinical trial registration allows the relevant stakeholders identify that a clinical trial exists. The systematic recording and monitoring of trial information within a registry goes some way to prevent and detect the selective publication and selective reporting of clinical research outcomes.

The large proportion of partially reported outcome data suggests that journal editors and reviewers need to more closely monitor and address this problem. Relevant reporting guidelines clearly advocate for complete reporting of outcome data and are endorsed by many journals [14]. However, routine checks of the essential items to be reported are often still not implemented in peer review and editorial procedures. As summarized in recent systematic reviews of non-publication and reporting biases [1] there is solid evidence confirming both areas of problem [2]. Future studies need to explore ways how the known underreporting of outcome data can be tackled effectively e.g. by creating incentives for complete and accurate reporting of outcome data from clinical trials and for making raw trial data more accessible.

## Ethical approval

No ethics committee opinion was required for this study.

## Supporting Information

S1 File Samples sizes and outcome summaries for all published and unpublished trials. (XLSX)
